# Four-Year-Olds Use a Mixture of Spatial Reference Frames

**DOI:** 10.1371/journal.pone.0131984

**Published:** 2015-07-02

**Authors:** James Negen, Marko Nardini

**Affiliations:** Department of Psychology, Durham University, Durham, County Durham, United Kingdom; Centre de Neuroscience Cognitive, FRANCE

## Abstract

Keeping track of unseen objects is an important spatial skill. In order to do this, people must situate the object in terms of different frames of reference, including body position (egocentric frame of reference), landmarks in the surrounding environment (extrinsic frame reference), or other attached features (intrinsic frame of reference). Nardini et al. hid a toy in one of 12 cups in front of children, turned the array when they were not looking, and then asked them to point to the cup with the toy. This forced children to use the intrinsic frame (information about the array of cups) to locate the hidden toy. Three-year-olds made systematic errors by using the wrong frame of reference, 4-year-olds were at chance, and only 5- and 6-year-olds were successful. Can we better understand the developmental change that takes place at four years? This paper uses a modelling approach to re-examine the data and distinguish three possible strategies that could lead to the previous results at four years: (1) Children were choosing cups randomly, (2) Children were pointing between the egocentric/extrinsic-cued location and the correct target, and (3) Children were pointing near the egocentric/extrinsic-cued location on some trials and near the target on the rest. Results heavily favor the last possibility: 4-year-olds were not just guessing or trying to combine the available frames of reference. They were using the intrinsic frame on some trials, but not doing so consistently. These insights suggest that accounts of improving spatial performance at 4 years need to explain why there is a mixture of responses. Further application of the selected model also suggests that children become both more reliant on the correct frame and more accurate with any chosen frame as they mature.

## Introduction

In order to move around the world, people must use different frames of reference. Different tasks or circumstances require specific frames. Children must learn to use the correct frame of reference for the various types of circumstances they encounter. The present study is a new look at the data from Nardini, Breckenridge, Burgess & Atkinson [1] focusing specifically on how the use of intrinsic spatial representations emerges in direct search tasks. Previous analyses showed that average error when a 4-year-old must only use the intrinsic frame is about what is expected from just guessing. The present paper applies a modelling approach to the existing data and shows that searches at 4 years were *influenced* by the intrinsic frame—just not to a point where it fully dominated the end behaviour. The key power of this new analysis is that we analyse the full spatial distribution of responses instead of just looking at the average error.

The method differentiates between three different ways of remembering where something is as you move around the world. First, you could encode the object’s location relative to your body e.g. “It’s down and to my left”. This kind of egocentric (self-based) representation is often a very salient one early in development [2]. It has the advantage of making it easy to locate or reach for an object from the same familiar viewpoint (*i*.*e*., as you face the front door from the middle of the room). However, it has the disadvantage of providing no direct basis for recall from any other place or direction.

Second, you could also encode where the object is in relation to a stable landmark, *e*.*g*. “It is near the big window.” This kind of representation has moved from being egocentric to allocentric (based on the world), and may include more or less rich detail about the surrounding environment. For example, simply saying “near the big window” is enough to restrict search to a sub-space within the room. Additional distance and angle information with respect to the window (and perhaps other stable landmarks in the room) can better pin-point locations. This kind of indirect coding with respect to external landmarks is commonly studied, for example (in animals) in the water maze [3]. We will use the term *extrinsic* to describe the coding of spatial relations with respect to large stable landmarks that are disconnected from a coherent object or array [4].

Third, you could encode the object’s location relative to other things that are attached to it (or at least, very likely to move when it moves). For example, the first author once came into his office to find that his cabinet had been moved to the other side of the room and had been turned 180°. In order to find my files, I needed to understand how they were related to other objects in my office, but I had to do so carefully; I could no longer use information about the larger room, but had to rely on information only about the cabinet (i.e. “the files are in the top left corner of the cabinet, *not* the right side of the room”). This kind of *intrinsic* spatial representation [4] is our focus in this paper. It is called *intrinsic* because it relies on the properties of a coherent array of things that move together, regardless of what happens outside of that array. (To be clear, both extrinsic and intrinsic frames are allocentric, meaning world-based.) Our central aim is to carefully characterize how and when the use of the intrinsic frame of reference emerges in direct search tasks.

### From Looking to Doing

There is evidence for egocentric, extrinsic and intrinsic frames of reference in infancy in passive looking-time tasks, but they only become used in active search tasks later. There is already evidence for basic egocentric coding of locations in newborns, who orient to visual [5], auditory [6], or tactile stimuli [7]. Infants can also encode the extrinsic frame of reference in looking-time tasks. For example, 6.5-month-olds notice changes to an object’s position on a table, even when these changes keep the relation between infant and object exactly the same [8]. This shows that they were already sensitive to the object’s place in relation to external landmarks (*e*.*g*. the table) and not only the egocentric relation between object and themselves.

Evidence for early intrinsic representations comes from the extensive “mental rotation” literature. The ability to relate an object to a rotated version of itself implies knowledge of the spatial relations within the object. Infants will look at a new object longer than a rotation of a familiar object, even if the new object is just a mirror of a familiar one, before their first birthday [9,10,11,12]. This shows that they have intrinsic representations that are powerful enough to tell apart objects just based on the spatial configuration of an identical set of features.

The present study is concerned with how children translate these early cognitive skills into the ability to physically find objects. Behaviourally, there is evidence that egocentric coding predominates in early actions. Piaget [13] suggested that children display an egocentric style of search very early on during bottle-feeding behaviour; if a bottle is turned upside down, they might suck on the wrong end as if they expected the useful end to remain in the same relation to their body. Infants trained to locate an experimenter on one side (e.g. their left) continue incorrectly to look to the same side after a translation and 180° rotation [14], even when correct and incorrect locations differed in highly distinctive visual cues [15]. There is evidence for improving abilities to process such rotations correctly, by taking into account their own movement and/or using external landmarks, from around 8.5 months [16].

By their second birthday, children can use extrinsic reasoning to supplement egocentric representations in direct search tasks. For example, Newcombe et al. [17] asked 16- to 24-month-olds to relocate an object buried in a sandbox from either the same side or after walking around to the opposite side. Children aged 22–24 months were more accurate when they could see additional landmarks around the room (see also [18]). There are also many studies where children are disoriented and must rely on external landmarks to re-orient themselves and find hidden objects back. Children show some level of competence at this sometime in the second year, depending on the task details [19], which has generated a great deal of theoretical debate regarding the underlying cognitive mechanisms [20,21].

Development of the intrinsic reference frame has been less studied. Clues again come from mental rotation, although a mental rotation task is not a search task. Success at adult versions of mental rotation tasks, in which participants explicitly judge whether a display is mirrored or not, is very slow to emerge (*see* [22] *for review*). Many 4–5 year olds still rely on strategies that do not involve correct rotation [23]. The present study further examines the use of the intrinsic frame in the context of a true direct search task.

To summarize our literature review: Looking time studies suggest that the egocentric, extrinsic, and intrinsic frames of reference are all applied in look-time tasks before the infants’ first birthday. However, much older children do not seem to use all three frames of reference in active search tasks: egocentric spatial coding is evident from birth, extrinsic frames appear before the second birthday, and intrinsic frames seem to emerge much later. Children start succeeding at active mental rotation tasks, which require intrinsic representations, sometime around the age of 4 years old [22], and the ability to use intrinsic frames in active search tasks emerge between the ages of 4 and 5 years [1].

Why might the age of 4 years provide a critical starting point for the emergence of intrinsic reference frames? A four-year-old has had extensive motor experience; has been using extrinsic representations for several years [17]; is experiencing a large upswing in executive function, notably the ability to suppress first impulses [24]; has probably begun producing simple spatial language like ‘left’ and ‘right’, but could still have considerable trouble using them correctly [25]; and can use a model representation of a space to find things under the right circumstances [26]. We now turn to the details of the present task and the analysis.

### The Task At Hand

**Fig 1:**
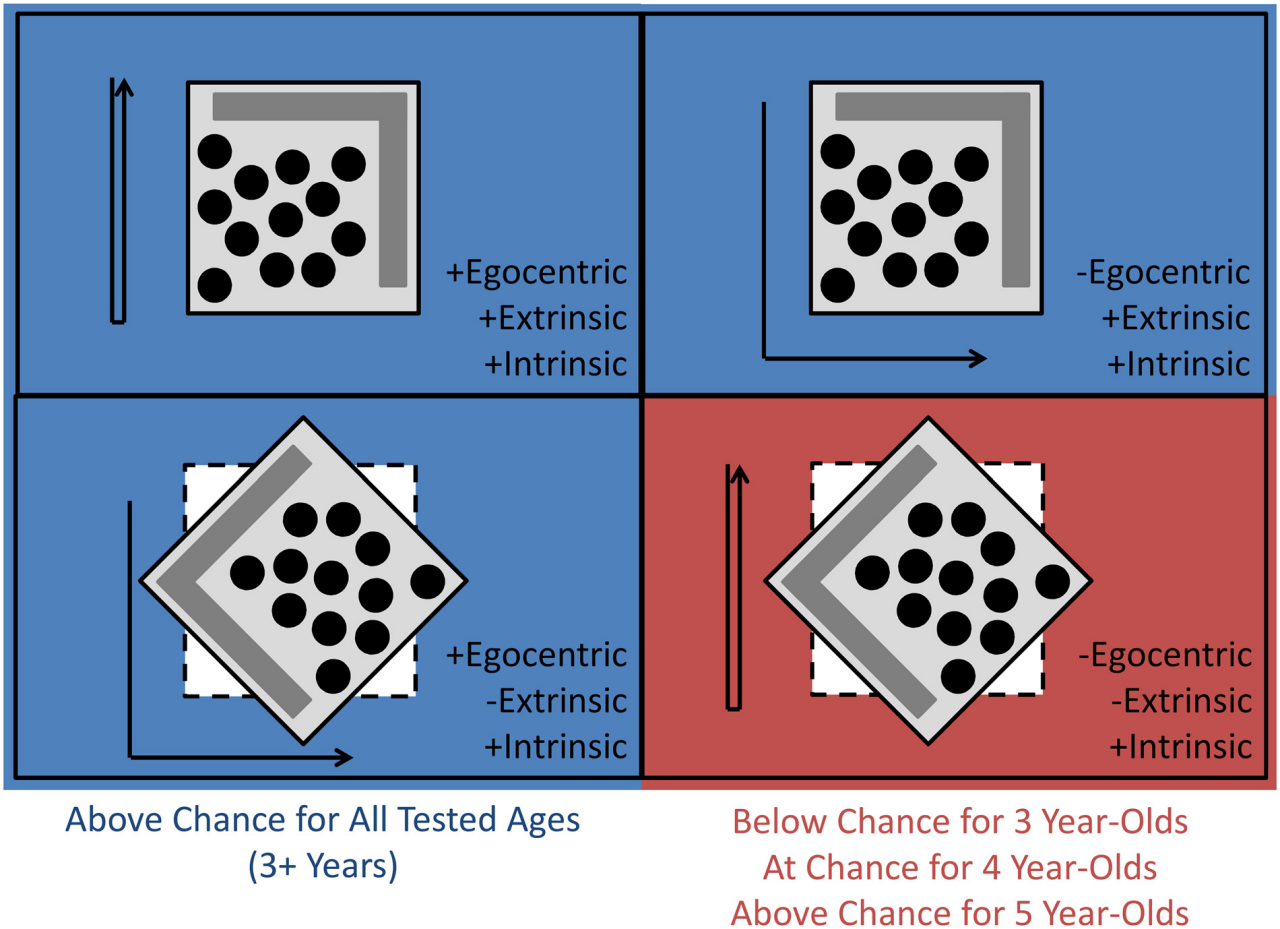
The ‘Town Square’ and the Task Conditions. The gray board is the movable array. The black circles are the hiding cups. The dark gray region includes useful landmarks (model houses and a toy frog and cat). The arrow shows how the child moves in each condition. The array was free to rotate within the room, but the spatial relations between the hiding places and landmarks on top of the board were never altered. After the child had moved and/or the array had moved, the child was asked to point to the cup where the toy had been hidden. The left two conditions allow for egocentric coding (where it is relative to you, called ‘body’ in [1]). The top two allow for extrinsic coding (where it is in the larger room, called ‘room’ in [1]). Our re-analysis here focuses on the way the 4 year-olds completed the bottom-right condition, which can only be done correctly by intrinsic cues (where it is relative to other members of the array, called ‘array’ in [1]). This is the only condition where performance (in terms of distance from correct location) was not above chance at some of the tested ages. The present study specifically asks what the 4-year-olds were doing that lead to their at-chance performance.

Nardini et al. [1] created a task that distinguished between children’s use of egocentric, extrinsic, and intrinsic representations. A small ‘town square’ (after [27]) was made on a movable board inside a larger room (Fig 1). There were 12 hiding places and several distinctive landmarks. Participants saw an experimenter hide a toy under one of the cups and had to retrieve it after different spatial manipulations. Participants either saw the board from the same viewpoint (allowing egocentric coding) or a different viewpoint (not allowing egocentric coding). The board either stayed in the same place within the room (allowing extrinsic coding) or was rotated within the room (not allowing extrinsic coding). None of the objects on top of the board ever moved. This meant that intrinsic coding (use of the objects on top–*i*.*e*. the spatial relations within the array) could solve all the conditions. Crucially, in the hardest condition, in which neither egocentric nor extrinsic coding worked, intrinsic reference was the only basis for recalling the location.

Nardini et al. [1] found that even 3 year olds could perform well above chance when the egocentric or extrinsic frame of reference was sufficient. In contrast, performance on the condition that could only be solved by the intrinsic frame was significantly above chance only for 5- and 6-year-olds. In that condition, three-year-olds would have done better by guessing; the way they searched was systematically incorrect (in line with use of egocentric and extrinsic coding, which predicts the wrong location on that condition). The four year olds, however, were neither closer on average to the correct intrinsic-cued location nor the incorrect egocentric/extrinsic-cued location than expected from chance guessing. What were they doing and what does it tell us about the emergence of intrinsic spatial representations at 4–5 years?

Here, a crucial detail of the original design must be understood. To dissociate the use of the different cues as much as possible, the array was turned 135° on the intrinsic-only trials, bringing the correct intrinsic-cued location nearly to the opposite side of the board from the incorrect egocentric/extrinsic-cued location. This means that true random guessing is not the only hypothesis that predicts an average error that is roughly equal to the chance-expectation. Specifically we tested three hypotheses that all predict this result, despite telling fundamentally different cognitive stories about spatial development.

### Random Guessing or Unrelated Cup Preference

These are the ‘null’ hypotheses. The first hypothesis is that children’s responses were actually random. The second is that they weren’t fully random, but they weren’t related to the spatial cues at all; they just happened to like some cups better than others. As it happens, there is already reason to doubt these before the modelling is applied: Among the 4 year-olds, 17/84 trials were at the correct cup in the intrinsic-only condition. This is more than would be expected from a 1/12 chance (since there were 12 cups), p = .0002. But the models generated by this hypothesis serve as useful benchmarks, and it is reassuring to see similar conclusions from different forms of analysis. Under this hypothesis, the 4 year olds understand that it isn’t right to use the egocentric/extrinsic cues but they don’t have any better way to proceed, so they either just guess or at least choose an attractive cup.

#### Cue-Combination

Children are failing to disregard the misleading egocentric/extrinsic cues and they combine them with the intrinsic cue. They see where all of these cues point and then point somewhere in between them, ‘averaging’ them spatially. This happens to lead to chance-expectation average distance error because of the fact that the cues were on nearly-opposite sides of the board; it tends to produce errors that are on average about half of the size of the array, just like true random guessing. This hypothesis is therefore a specific kind of weighted cue combination [28], *c*.*f*. [29]. Under this hypothesis, the 5 year old succeeds where the 4 year old doesn’t because (s)he understands that the various cues are simply too disparate to be reconciled and the egocentric/extrinsic cues should just be ignored.

#### Cue Mixing

Roughly half of responses are driven by the egocentric/extrinsic cues and the other half are driven by the correct intrinsic cues. This again happens to lead to chance-expectation average distance error because the cued locations were on nearly-opposite sides of the board; it produces many very small errors (attending to the correct cue) and many very large errors (attending to the misleading cues) which average to errors of about half the array size, just like true random guessing. On some trials, the child adopts the correct strategy—e.g. attending to intrinsic spatial relations to encode the location, and/or inhibiting the incorrect egocentric/extrinsic cues when responding—and on other trials that same child fails to do so. This should prompt further investigation into the exact nature of the deficit that prevents them from using the correct strategy more often.

With this last model, we also have the opportunity to see if the rise in performance with age is due to using the correct frame more often, becoming more accurate at choosing the cup that the chosen frame indicates, or both.

## Modelling Method

To test these data, we form a series of models and fit them to the spatial distribution of children’s responses. We provide each model with a full generative specification; we make it possible to work out the exact probability of choosing each cup on each trial if the parameters are set, and then we give each parameter a proper prior distribution that integrates to 1. This allows us to do away with the typical framework of null hypothesis testing and instead just see which model’s predictions are better matches to the actual data. We will compare the models by Deviance Information Criterion (DIC) [30] and Bayes factors [31,32]. Crucially, both methods account for the spread in each model’s predictions. DIC does so explicitly by estimating the number of ‘effective parameters’ and adding that as a penalty to the final score. Bayes factors do so implicitly by looking at how well the data are fit from every point in the prior parameter space, rather than just using the parameters settings that fit the best (*c*.*f*. a *t*-test, where the best-fit means and standard deviations are used to compare the null and the alternative hypothesis). The upshot is that the analogue to a ‘null’ hypothesis in frequentist hypothesis testing can actually be preferred, rather than just failing to reject it. (We will also check that this is working by generating random data and making sure that the simpler Random Guessing hypothesis is selected by the analysis method.) To review a model-fitting Bayesian approach to data analysis, see [33,34].

These models are applied to data from the 21 4-year-olds (11 male) in the intrinsic-only condition. Distances were coded as proportions of the length of one side of the moving board. The models are designed to take the hypotheses above and turn them into mathematical specifications that can be tested with standard Bayesian methods. Models were developed in WinBUGS [35] and code appears in S2 File. Since this was a re-analysis, no new consent was gathered from the participants (though full written consent was gathered for [1] in line with the Ethics Committee at University College London in the Psychology Department). The data themselves appear in S3 and S4 Files and S1 Fig.

### Random Guessing and Unrelated Cup Preference Models

#### Random Guessing

**Fig 2:**
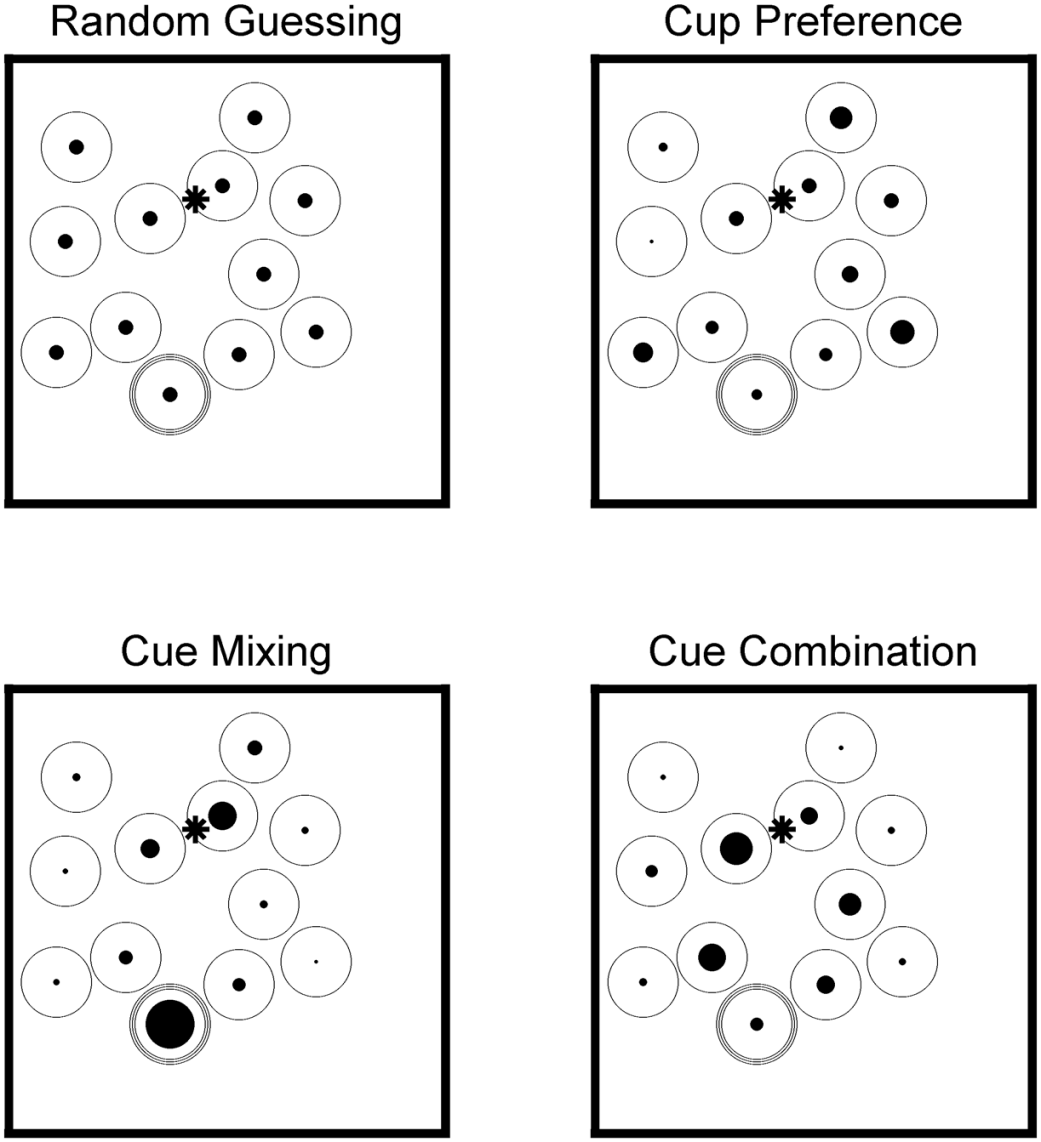
Example predictions from the 4 models. The triple rings indicate the correct response, which is always indicated by intrinsic cues. Larger fills are more probable. The egocentric/extrinsic-cued location is marked with a star (near center, about ¾ up). The Random Guessing Model just assigns all cups an equal probability. The Cup Preference Model allows for some cups to be preferred over others in a way that is independent of the actual target or other cues (e.g. the cup by the frog is attractive). The Cue Mixing Model says that responses will be clustered around the correct target (triple rings) and the egocentric/extrinsic-cued location (star). The Cue Combination Model says that responses will cluster around the midpoint between the correct target (triple rings) and the egocentric/extrinsic foil (star). All four of these models predict that the average distance error will be roughly equal to half the size of the array, which corresponds to the original result in Nardini et al. [1].

Under this model (Fig 2, Upper Left), the probability of a child choosing a given cup on a given trial is always 1/12, since there were 12 cups. It has no parameters and thus no priors.

#### Unrelated Cup Preference

**Fig 3:**
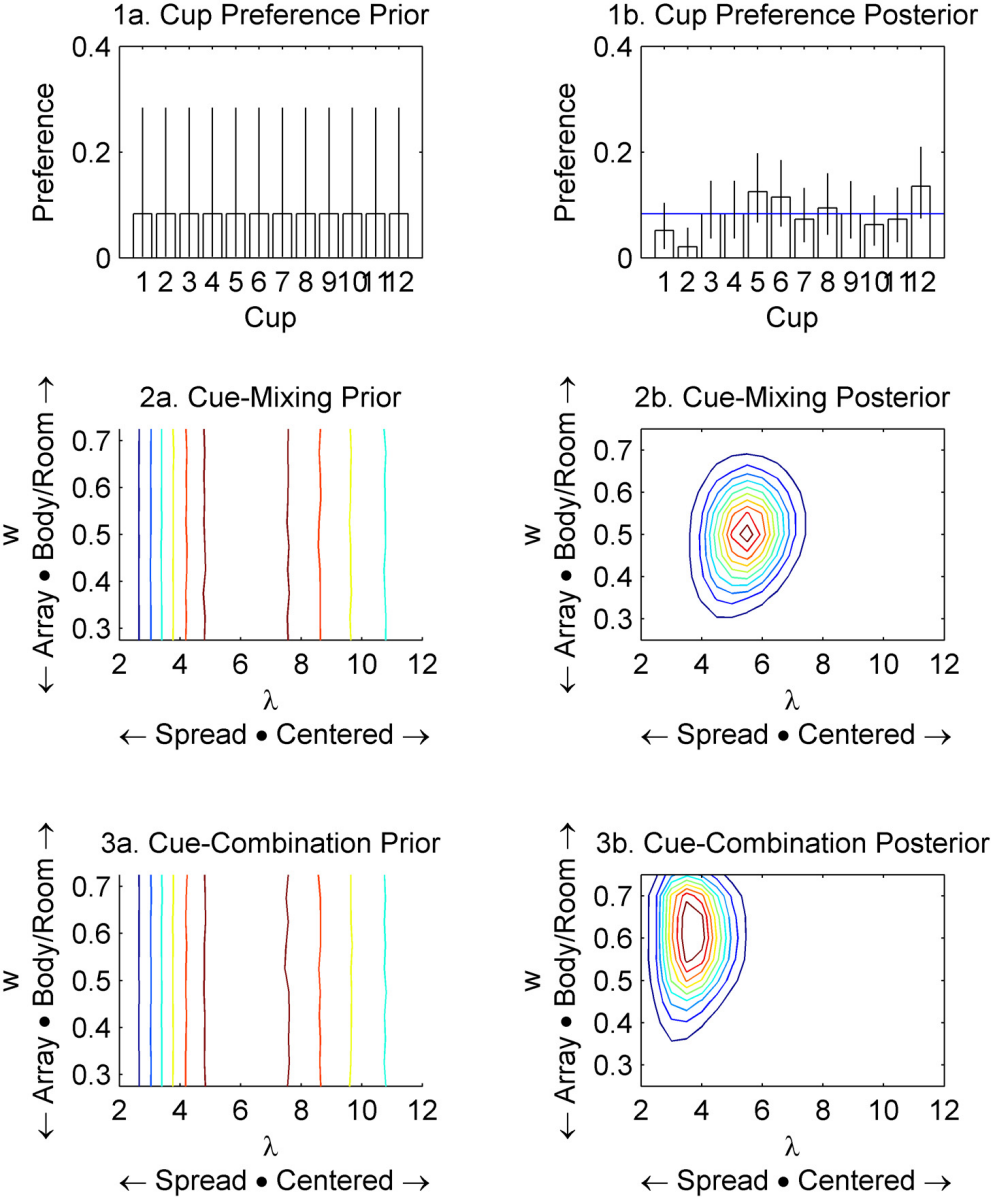
Prior (left) and Posterior (right) distributions of each model’s parameters. Bayes factors comparisons can be problematic if the posterior is radically outside the central coverage of the prior, though *c*.*f*. [36]. In this case, this does not appear to be an issue. 1b. The cup preference model does not strongly suggest than any cup has above-chance preference (blue line). Error bars are 95% CI. 2b. The Cue-Mixing Model fits fits a mixture that is about 50% of each cued location, which is needed to correctly predict that mean error will be at about the chance expectation. 3b. The Cue-Combination model fits a higher spread than the Cue-Mixing Model.

Under this model (Fig 2, Upper Right), each cup *i* has a response probability P_i_ across all trials and participants, regardless of cues. This model simply says that, for example, the cup by the frog is an attractive response. This model is given a prior of Dirichlet(1,…1). This prior is a standard choice for situations where we have nominal data and little reason to expect any particular response to be especially frequent. Fig 3 (left) shows the priors for each model graphically. The mean of the prior on each P_i_ is 1/12, with a 95% CI that stretches from 0.23% to 28.49%. This prior is uninformative in the sense that (1) no particular response is favoured and (2) any combination of 12 percentages that add up to 100% is supported. Priors act as expressions of what we think might be more or less likely before we look at the data, and they serve to make Bayes factors comparisons work by forcing each model, no matter how simple or complex, to be able to generate synthetic data to be compared with the actual data.

### Cue-Combination Model

This model (Fig 2, Lower Right) draws a line between the correct intrinsic-cued location and the incorrect egocentric/extrinsic-cued location, picks a point near the middle of that line, and then assigns probability to each cup based on distance away from that point. For example, if the target is now at the bottom left, and the egocentric/extrinsic-cued location is in the top left, then the expected response will be near the middle left. There is a weight parameter *w*, where *w* = .5 would be the middle of the line and *w* = .75 would be 75% of the way towards the actual target. This parameter is restricted to .25<*w* < .75 so that it must always combine some of each cue, since this is the only way to predict average error that is near the chance-expectation, and it also makes sure that the model can be interpreted as intended. There is also a precision parameter λ that controls how far responses are spread from the central point by exponential decay. Specifically, the probability of choosing a given cup is proportional to e^(-λd), where d is the distance from the central response location to the cup. The priors are Gamma(3, ½)+2 for λ and Uniform(.25, .75) for *w*. This gamma prior has support from 2 to positive infinity and a mean of 8 (= 3 / ½ + 2). For reference, Fig 3 is drawn with *w* = .5 and λ = 8. Values of λ below 2 are excluded because they spread the probability so much that it becomes almost equivalent to the Random Guessing model (i.e. the probability of choosing each cup is only a few percentage points off of the probability assigned by the Guessing model).

### Cue-Mixing Model

This model (Fig 2, Lower Left) says that children will point near the intrinsic-cued cup with probability *w* and near the egocentric/extrinsic-cued location with probability (1-w). This is also restricted to .25<w < .75 for the same reasons. There is also a λ parameter with the same function: controlling how far responses are spread from the chosen location. Again, the probability of choosing a given cup is proportional to e^(-λd), where d is the distance from the cued location to the cup. For simplicity we assume that all cues have equal λ. The priors are again Gamma(3, ½)+2 over the common λ and Uniform(.25, .75) over *w*. The crucial empirical difference between the Cue Combination and Cue Mixing model is that the Cue Combination model predicts a unimodal distribution of responses (one mode between the two cues), but the Cue Mixing model predicts a bimodal distribution (one mode at each of the two cues).

## Results

4 independent chains of 25,000 samples were drawn in WinBUGS [35]. $\hat{R}\;$ statistics [37] are all within .001 of 1, which is considered good. Prior and posterior parameters for each of the models are shown in Fig 3. The posterior for the Cue Mixing model is the most directly sensible. It has a strong concentration at *w* = .5, where it strongly predicts the original result, though the Cue Combination model has some posterior density there as well. The Cue Mixing model also has a higher average posterior λ than the Cue Combination model (meaning that it makes stronger predictions). In contrast, the Cup Preference model has not found any cups clearly above the 1/12 line expected from a uniform preference. This does not bode well for the model’s ability to assign high probability to any given response. It also makes the substantive interpretation awkward, since the ‘Cup Preference’ model is not actually finding any clear preference for any particular cup.

We compare the fit of the models in two ways (Table 1), both of which favour the Cue-Mixing model. The first method is Deviance Information Criterion (DIC) [30]. This method computes the average deviance, which is -2 times the summed log-probability of the data, plus the number of ‘effective parameters’, meant to be an estimate of the flexibility and complexity of the model. The model is given a higher score for worse fit or more parameters (so lower DIC is better). A difference of 3 to 7 is generally considered large [30]. In our analysis, Cue Mixing comes out with the lowest DIC (Table 1). Cue Combination is behind by 16.87.

**Table 1:** Results of the 4 models being fit to the data.

Model	DIC	Prior Weight^b^	Posterior Weight^b^	Bayes Factor
Random Guessing	417.46^a^	.0087	.2634	30.2075
Cup Preference	1484.30	.9906	.2698	.2723
Cue Combination	409.70	.0007	.2208	335.1674
Cue Mixing	**392.83**	5.04*10^−8^	.2460	**4,873,849.4522**

Lower DIC is better, and higher Bayes factor is better, so Cue Mixing has the best score in both metrics. Bayes factors are ± 0.96% (95% CI).

^a^DIC is perhaps not properly defined for a model with no variable parameter space. If the choice probability is considered a parameter, then it has no variance, and thus $\;\overset{-}{D}\;=\;D(\overset{-}{\theta })$. Reported here is simply the deviance, or equivalently, the DIC with *p*_D_ = 0. The values of *p*_D_ were 0, 9.49, 1.13 and 1.81, respectively.

^b^These are not independent metrics. These numbers are presented to show that the priors have been adjusted to make the posterior roughly equivalent, since we need a large number of samples for each model in order to make an accurate estimate of each Bayes factor. The percentage error in estimated Bayes factor for each of the 4 models is expected to be very similar, within 0.15%, when we have 100,000 samples and these posterior weights.

The second method is through a Bayes factor [31,32]. This method is often preferred because it is the most direct way of performing model selection: it tells you the probability that a model is correct given the data and an uninformative prior. It can do things that many other methods cannot, such as actively favour fitting a line with 3 change points over a line with 4 [32], rather than simply failing to reject the null 3-point hypothesis. In some cases it is demonstrably better at choosing the correct model than DIC [38]. The first issue is that it often cannot practically be computed in many cases, especially when hierarchical modelling is involved. However, we are able to estimate it here through WinBUGS.

The second issue is that priors are a factor in the calculations. A model will generally do better if its prior is adjusted so that it has higher prior probability at points where it has better fit to the data. This could be potentially abused when the likelihood has little influence and the priors don't reflect the actual hypothesis that they are supposed to instantiate (or are simply so complex/vague that they can't be evaluated). We show in S1 File that the winning model still wins even after it suffers this abuse. We have also laid out what each parameter does in each model and we have given each a common prior with a clear interpretation. The full priors and posteriors are available in Fig 3 to show that the priors are neither complex nor vague. We have given *w* a prior that reflects the known outcome (mean error at chance expectation) and we have given λ a prior that reflects a plausible range of precision for 4-year-olds without allowing it to degenerate into another random guessing model.

Bayes factors were estimated by combining all 4 models into a single larger model with a model choice parameter *m*. When *m* = 1, the model behaves like the first model, assigning each cup a 1/12 chance on all trials. When *m* = 2, it behaves like the second model, choosing cups based on cup preference. This goes on through all 4 models. The variable *m* can then be sampled just like any other parameter [31]. The ratio between the posterior and the prior can then be interpreted as a Bayes factor, a measurement of how much better each model fits the data as you integrate over its prior. A factor of over 100 is considered “decisive” [31]. As Table 1 shows, the Cue Mixing model has the highest Bayes factor, and this was more than 1000 times higher than all other models. In summary, the Cue-Mixing Model should be preferred because it has a sensible posterior that is peaked at *w* = .5, where it predicts the previous results; it has the lowest DIC by 16.7; and it has the highest Bayes factor by over 1,000.

S1 File presents a number of additional analyses that were done to support the validity of the main analysis done here, described briefly: (1) we generated data from the Random Guessing model and showed that the analysis method favours that model when given those data; (2) we computed the model comparisons with all of the ages, adding AIC and BIC metrics, and obtained expected results; (3) we looked for possible heterogeneity in the 4-year-olds but did not find any evidence; (4) we used an alternative decay function and found similar results; (5) we empirically fit the priors to the Cue Combination model and found that it was still disfavoured by over 1,000:1.

### Parameter Changes by Age

The Cue Mixing model has another interesting feature, namely that it formally separates the contributions of two sources of error. A low *w* shows that children are frequently using the incorrect frame. A low λ shows that children have poor precision with whichever frame they have chosen. We looked at parameter estimates for each group with the Cue Mixing model. For this comparison, we opened up w to the range of 0<w<1, since this makes more sense for the youngest and oldest groups. In S1 File, we show that Cue Mixing is the preferred model for 4-, 5-, and 6-year-olds; an Egocentric/Extrinsic-Only model where *w* = 0 is preferred for the 3-year-olds. But we did not find any evidence that they go through a Cue Combination phase, a Cup Preference phase, or a Just Guessing phase, so this model is a reasonable choice for parameter estimates.

**Fig 4:**
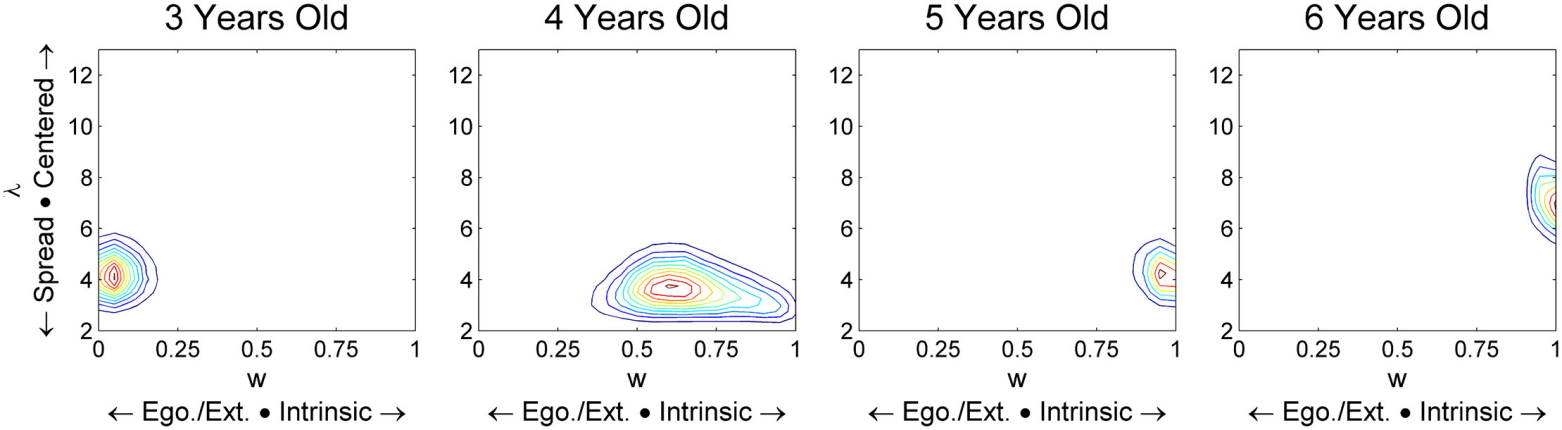
Parameter estimates from the Cue Mixing model at each age. This model has a separate parameter for the frame of reference being chosen (x axis) and the concentration of responses around the place indicated by that frame (y axis). Both are seen to improve with age here.

Fig 4 shows the posterior distributions of w and λ. Leftwards indicates heavier use of the egocentric/extrinsic frame. Rightwards indicates heavier use of the intrinsic frame. Downwards indicates more spread in the responses around the two centers. Upwards indicates higher concentration of responses. The posterior estimates move both rightwards and upwards as age increases. This is interesting since it suggests that the developmental process at play is not just becoming more reliant on the intrinsic frame, nor is it just becoming more precise in terms of spatial memory using a given chosen frame. There appear to be separate contributions of both.

Interestingly, the same general trend is seen when we look at parameter estimates from the Cue Combination model over age (S5 File).

## Discussion

Nardini et al. [1] hid a toy in one of 12 cups in front of children, turned the array when they were not looking, and then asked them to point to the cup with the toy. Previous analyses noted that the average distance error from 4 year olds was roughly as expected if they were guessing. Several possibilities could lead to this result, which we compared using Bayesian model selection methods. New analyses show that random guessing behaviour cannot accurately predict the spatial distribution of responses. Neither can simply picking a preferred hiding location repeatedly. Neither does spatially ‘averaging’ the correct intrinsic cues and the incorrect egocentric/extrinsic cues, pointing to a cup that is between the two cued locations (see Fig 2, lower right for an example). Instead, children in the transitional period rely on egocentric/extrinsic cues about half of the time and intrinsic cues the other half. This suggests that the intrinsic representations are actually present at four years old, though they have still not fully taken over behaviour. This in turn allows us to point towards likely explanations for what is driving the improvement in performance in this age range.

There could be many ways to explain why this mixing of egocentric/extrinsic and intrinsic-cue reliance happens at 4 years and why it eventually stops, but one explanation stands out as especially simple and intriguing: during the 4^th^ year, the improved performance on this kind of spatial task does not depend on the development of spatial cognition *per se* at all. The underlying spatial representations are already present, but some frames are easier and more salient to use, so children have an impulse to use them first. In particular, the egocentric cues are easiest, followed by extrinsic cues, then intrinsic cues. Under this kind of explanation, children at 3 years old have developed enough inhibition skills to inhibit an egocentric response but not an extrinsic response, whereas children at 5 years old can inhibit both. This fits nicely with many other findings that show dramatic increases in other measures of executive function in this age range [24]. It also makes sense in light of the fact that infants seem able to mentally rotate objects, which suggests that competence using the intrinsic frame is present, but somehow not always expressed. The next step in testing this hypothesis is measuring individual differences in executive function to see if these differences predict performance on the task. There is perhaps even the possibility that the three year olds would be capable of the task if it did not involve the same kind of cue conflicts; the difference between the intrinsic cues and the extrinsic cues is an artifact of experimental design rather than a desired feature.

Additionally, or alternatively, differences may not be only at the recall stage, which requires participants to correctly select and inhibit frames of reference, but also at the initial encoding stage. The development at 4 years may be in the choice to attend strategically to the hiding location in terms of its place on the board (intrinsic cues) rather than its place in the room or relative to the body. Further research is needed to test this hypothesis.

Results also suggest that the development from 3 to 6 years is both (a) learning to use the intrinsic frame more often when it is needed and (b) being more precise with whichever frame is being used. The Cue Mixing model was fit to the responses from each year. The posterior estimates suggest that both *w* (the chance of using the intrinsic frame) and λ (concentration around the place cued by the chosen frame) increase steadily with age.

Note that strong reliance on the intrinsic cues requires an understanding of the situation that is separate from having a classic metric ‘map’—in one very specific way, it is actually more advanced. A full metric representation of the space at the time of encoding, including a rich and accurate list of angles and distances between things, is not exactly what is needed. In order to correctly calculate the desired object’s new place, it must also be understood that some of these spatial relations have remained consistent and some have not. In particular, the local spatial relations between cups in the array have remained constant, but they have all systematically shifted in relation to their place in the larger room. One must filter out irrelevant extrinsic cues and represent relevant intrinsic cues in a way that allows the correct spot to be found again. In other words, the task requires a kind of flexibility that a classic map does not offer. On the other hand, overall success at the task does not always require a specifically-metric strategy e.g. encoding that a toy was nearest the frog would be enough on some trials, even without noting the exact distance or angle. In such cases, a map is both more powerful than needed (with exact angles and distances) and not powerful enough (lacking flexibility).

Note also that we have largely left aside the issue of “dead reckoning” or “path integration”, when an organism keeps track of where something is relative to itself and updates that memory by the perception of its own movement [17]. For example, if you stand by an object, close your eyes, take two big steps forward, turn 90° left, and take 1 more step, you can probably tell very quickly that the object is to your left and slightly back, even without looking. The way the present experiment was done, path integration cues were always redundant with extrinsic cues. Disrupting this connection requires that the child be disoriented somehow, which was not done. For shorthand we simply talked about the extrinsic cues, but it is also true that children may have been using path integration instead of looking at the larger landmarks. The counter-argument is that path integration errors accumulate rapidly, especially in small children, so it’s very possible that using the actual room landmarks would be less noisy. Humans can do some path integration, but we are not as accurate as animals that rely on it heavily for food foraging [39].

In conclusion, the original results [1] should be interpreted in a specific way—on average, the 4 year-olds were not closer to the correct location than you would expect just from guessing, but that does not mean that they actually were just guessing. The design of the experiment allows for several different strategies to all create similar average error. However these different strategies create different spatial distributions of responses, which we examined here. Our new analyses suggest that children in this age range are actually using the intrinsic frame about half of the time in a direct search task, but actually being actively misled by the egocentric/extrinsic frames on the other half. It is possible that a key developmental change is in executive function, particularly inhibition. It is also possible that there are changes in the reference frames used during the encoding process. Both these explanations fit the available evidence, but require direct testing in future studies.

## Supporting Information

S1 Fig Positions Diagram. (GIF)

S1 File Analysis Checks. (DOCX)

S2 File WinBUGS Code for the Main Analysis. (DOCX)

S3 File Raw Data. (XLSX)

S4 File Data Notes. (TXT)

S5 File Parameter Estimates by Age with the Cue Combination Model. (DOCX)
