# Correction: Four-Year-Olds Use a Mixture of Spatial Reference Frames

**DOI:** 10.1371/journal.pone.0134973

**Published:** 2015-07-31

**Authors:** James Negen, Marko Nardini

Fig 4 incorrectly appears as a duplicate of Fig S5. The authors have provided a correct version of Fig 4 here.

**Fig 4:**
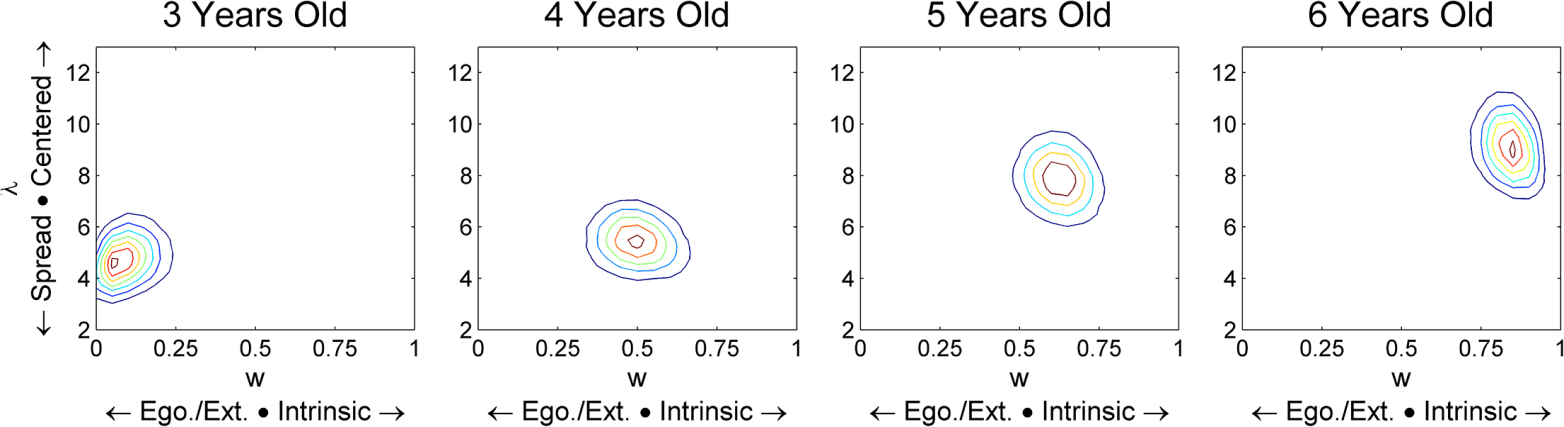
Parameter estimates from the Cue Mixing model at each age. This model has a separate parameter for the frame of reference being chosen (x axis) and the concentration of responses around the place indicated by that frame (y axis). Both are seen to improve with age here.
