# Supplementary figures and images for: Four-Year-Olds Use a Mixture of Spatial Reference Frames
Source: PLoS One. 2015 Jul 2;10(7):e0131984. doi: 10.1371/journal.pone.0131984 (PMC4489865; doi:10.1371/journal.pone.0131984)

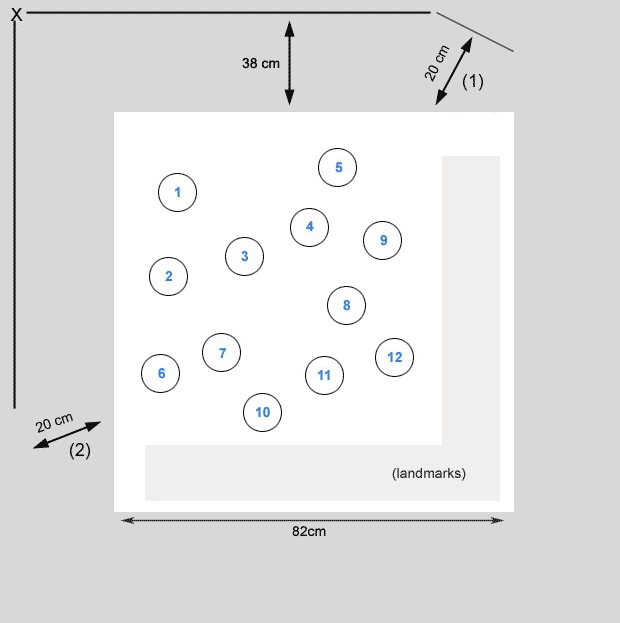

Supplement: S1 Fig — (GIF) [file pone.0131984.s001.gif]
