# Supplementary material for: Four-Year-Olds Use a Mixture of Spatial Reference Frames
Source: PLoS One. 2015 Jul 2;10(7):e0131984. doi: 10.1371/journal.pone.0131984 (PMC4489865; doi:10.1371/journal.pone.0131984)
Supplement: S1 File — (DOCX) [file pone.0131984.s002.docx]

# S1: Analysis Checks

We performed several additional analyses to check that the analysis method is working correctly and that it produces other results as expected. First, we generated datasets from the Random Guessing and Cup Preference models to make sure they would be selected. The Random Guessing dataset was generated by randomly re-pairing responses with trials. When the Bayes factor analysis was re-run, Random Guessing won with a Bayes factor of 857.92 (over 8.61 for Cup Preference, .46 for Cue Mixing and .10 for Cue Combination). The Cup Preference dataset was just a list of the following choices, made without looking at the actual target: cup 1 for the first 29 trials, and then cup 2-12 each 5 times. Here the Cup Preference model won with a Bayes factor of 1.07*10^10^ (over 1.07*10^6^, 1.94, and .35). This confirms that the method would prefer one of the two ‘null’ models if the data were actually just random guessing or just preference for a particular cup.

Second, we applied the models to the full age range with additional analysis modalities to see if it produces sensible results. Previous analyses suggested that the 3-year-olds mainly use the egocentric/extrinsic frame on the hardest trials and the 6-year-olds mainly use the intrinsic frame. For the analyses with other age ranges, we formed two additional models. The Egocentric/Extrinisic Only model is the same as Cue Mixing with *w*=0. The Intrinsic Only model is the same as Cue Mixing with *w*=1. We also calculated AIC (Akike Information Criterion) and BIC (Bayesian Information Criterion) for each model at each age range. We used a parameter count of 0 for Random Guessing, 12 for Cup Preference, 2 for Cue Mixing and Combination, and 1 for the Egocentric/Extrinisic-Only and Intrinsic-Only. To approximate the maximum likelihood, we calculated the likelihood for all values of *w* from 0 to 1 in increments of .01 and all values of λ from .01 to 10 in increments of .1. We used the maximum from this procedure as the maximum likelihood in the AIC and BIC calculations. Tables 1-4 show the results.

## Table S1-A. Results for 3 Year Olds.

|  |  |  |  |  |  |
| --- | --- | --- | --- | --- | --- |
| Model | AIC | BIC | DIC | Posterior Weight | Bayes Factor |
| Random Guessing | 342.92 | 342.92 | 342.92 | .169 | 6.5154e+08 |
| Cup Preference | 348.67 | 375.48 | 343.73 | .150 | 3.4134e+07 |
| Cue Combination | 320.39 | 324.85 | 329.46 | .165 | 2.8582e+10 |
| Cue Mixing | 319.99 | 324.46 | 324.37 | .167 | 8.2371e+11 |
| Ego. / Ext. Only | **318.39** | **320.62** | **318.08** | .180 | **6.1204e+13** |
| Intrinsic Only | 345.01 | 347.24 | 374.75 | .167 | 1.6700e-01 |

## Table S1-B. Results for 4 Year Olds.

|  |  |  |  |  |  |
| --- | --- | --- | --- | --- | --- |
| Model | AIC | BIC | DIC | Posterior Weight | Bayes Factor |
| Random Guessing | 417.46 | 417.46 | 417.46^a^ | .167 | 58.8878 |
| Cup Preference | 424.75 | 453.92 | 420.24 | .155 | 0.5389 |
| Cue Combination | 410.82 | 415.68 | 409.70 | .169 | 640.8476 |
| Cue Mixing | **393.21** | **398.07** | **392.83** | .164 | **9,620,863.2576** |
| Ego. / Ext. Only | 416.59 | 419.02 | 420.81 | .172 | 0.2426 |
| Intrinsic Only | 408.82 | 411.25 | 409.67 | .173 | 151.7760 |

## Table S1-C. Results for 5 Year Olds.

|  |  |  |  |  |  |
| --- | --- | --- | --- | --- | --- |
| Model | AIC | BIC | DIC | Posterior Weight | Bayes Factor |
| Random Guessing | 337.95 | 337.95 | 337.95 | .194 | 3.0492e+03 |
| Cup Preference | 348.06 | 374.69 | 342.97 | .171 | 2.0016e+01 |
| Cue Combination | 310.87 | 315.32 | 324.06 | .188 | 2.5724e+05 |
| Cue Mixing | **284.57** | **289.01** | **284.10** | .194 | **8.9393e+14** |
| Ego. / Ext. Only | 339.95 | 342.17 | 351.25 | .049 | 4.960e-02 |
| Intrinsic Only | 308.87 | 311.10 | 308.61 | .197 | 2.2377e+09 |

## Table S1-D. Results for 6 Year Olds.

|  |  |  |  |  |  |
| --- | --- | --- | --- | --- | --- |
| Model | AIC | BIC | DIC | Posterior Weight | Bayes Factor |
| Random Guessing | 337.95 | 337.95 | 332.98 | 4.4000e-04 | 8.800e-04 |
| Cup Preference | 348.06 | 374.69 | 329.15 | 2.000e-05 | 4.000e-05 |
| Cue Combination | 310.87 | 315.32 | 289.11 | .34 | 1.0308e+05 |
| Cue Mixing | **284.57** | **289.01** | **240.23** | .32 | **1.066e+16** |
| Ego. / Ext. Only | 339.95 | 342.17 | 362.19 | **--** | **--**^a^ |
| Intrinsic Only | 308.87 | 311.10 | 247.16 | .34 | 2.2459e+15 |

^a^Too low to estimate using 32-bit floating-point precision.

The results here are sensible. For every age, the model that is in first place by any measure is in first place by all measures. The Egocentric/Extrinsic Only model is preferred by every method for the 3-year-olds. The two additional methods (AIC and BIC) agree with the main results for the 4-year-olds, also by a margin greater than 10. For the 5-year-olds, there is a preference for Cue Mixing that is even stronger, with Intrinsic Only in second place and Egocentric/Extrinsic Only falling to last place in terms of Bayes factor. For the 6-year-olds, there is a marginal preference for Cue Mixing over Intrinsic Only. This last result may be surprising, but even the 6 year olds were not at ceiling by mean error.

Third, we examined the heterogeneity in terms of *w* that is present in the data. So far, we have argued that the 4 year-olds as a group were picking the intrinsic-cued cup half of the time and the egocentric/extrinsic-cued location on the other half. The results could also be obtained if half of the children always used the intrinsic frame and the other half always used the egocentric/extrinsic frame. Each child made 4 responses. We counted the number of responses that are nearer the intrinsic*-*cued location for each child. If there is extreme heterogeneity, this should be mostly 0 or 4, with relatively few near 2. Figure S1 shows that this is not the case.

**Figure S1-A. Number of Intrinsic-Side Choices per Participant.** Only 2/21 of the 4-year-olds gave all egocentric/extrinsic-side choices. None gave all their responses closer to the intrinsic side. If most children gave all one or the other, it would show that the models are inappropriate because of the heterogeneity in the data.

Fourth, we assessed sensitivity to the form of the distance decay function. For simplicity in the main analysis, we used an exponential decay, specifically e^(-λ*d). This is a convenient function because it is not infinite at its mode, has its mode at zero, is monotonically decreasing, scales easily, never fully reaches zero, and computes quickly. We checked to see if the results are the same if we use another function with similar properties, specifically the pdf of the Cauchy distribution: 1 / ( π * λ * (1 + (d/λ)^2 ) ). This function has a 'heavy tail' and thus may be able to fit outliers better than the exponential. Bayes factor results are similar for the 4-year-olds, with Cue Mixing at 103,570 (versus 414.69 for Cue Combination, .25 for Cup Preference and 29.55 for Random Guessing). This suggests that the results are not due to the idiosyncrasies of the exponential decay function.

Fifth, we assessed sensitivity to the exact priors for the Bayes Factor comparison. We formed new priors on *w* and λ that (a) have modes that are very near the peak likelihood of the second-place Cue Combination model and (b) have smaller variance. Specifically we gave *w* a Beta(10,6) and λ a Gamma(3,1), with respective modes at .6429 and 2. This still leaves Cue Mixing at over a 1,000:1 advantage, even with the priors over-fit to the Cue Combination model. This suggests strongly that the Cue Combination model is not disfavoured because of low prior probability in its region of highest likelihood.
