# Supplementary material for: Four-Year-Olds Use a Mixture of Spatial Reference Frames
Source: PLoS One. 2015 Jul 2;10(7):e0131984. doi: 10.1371/journal.pone.0131984 (PMC4489865; doi:10.1371/journal.pone.0131984)
Supplement: S2 File — (DOCX) [file pone.0131984.s003.docx]

## S2: WinBUGS Code for the Main Analysis

model{

*#Priors for the models with w and lambda.*

for (i in 1:nModels){

*#lambdaTMP is a temporary variable used for sampling since winBUGS does not have a shifted gamma distribution. dgamma is the gamma distribution. The parameters are shape, rate.*

lambdaTMP[i] ~ dgamma(3,.5)

lambda[i] <- lambdaTMP[i] + 2

*#dunif is the uniform distribution.*

w[i] ~ dunif(.25,.75)

*#Alpha here is the prior probability of each model.*

Alpha[i] <- alpha[i] / sum(alpha[])

}

for (i in 1:nCups){

*#This instantiates a Dirichlet(1,…,1) distribution by sampling from an exponential and then dividing by their sum.*

*#Preference[i] is the probability of choosing cup i under the cup preference model.*

preference[i] ~ dexp(1)

Preference[i] <- preference[i] / sum(preference[])

}

#dcat is a categorical draw.

theModel ~ dcat(Alpha[])

for (i in 1:M){ #M is the number of responses.

*#target[i,1] is the x-position of the actual target.*

*#target[i,2] is the y-position of the actual target.*

*#ego[i,1] and ego[i,2] are the x and y positions of where it should be by egocentric/extrinsic logic.*

*#center[i,1] and center[i,2] are the center point between the two for the Cue Combination model.*

center[i,1] <- w[4] * target[i,1] + (1-w[4]) * ego[i,1]

center[i,2] <- w[4] * target[i,2] + (1-w[4]) * ego[i,2]

for (j in 1:nCups){

*#respondFrom is the position in the testing space where the child had to respond from.*

*#cupsXY is the X/Y position of the 12 cups from the two perspectives.*

*#These calculations here find the distance from each cup to the target / egocentric projection / center between them by the Pythagorean theorem.*

distanceTarget[i,j] <- sqrt(pow(cupsXY[respondFrom[i],j,1]-target[i,1],2)+pow(cupsXY[respondFrom[i],j,2]-target[i,2],2))

distanceEgo[i,j] <- sqrt(pow(cupsXY[respondFrom[i],j,1]-ego[i,1],2) + pow(cupsXY[respondFrom[i],j,2]-ego[i,2],2))

distanceCenter[i,j] <- sqrt(pow(cupsXY[respondFrom[i],j,1]-center[i,1],2) + pow(cupsXY[respondFrom[i],j,2]-center[i,2],2))

*#These calculations turn the distances above into probabilities by the exponential decay formula.*

for (k in 1:nModels){

*#Ttmp, Etmp, and Ctmp are temporary variables used to normalize the probability so it sums to 1.*

Ttmp[i,j,k] <- exp(-lambda[k] * distanceTarget[i,j])

Tp[i,j,k] <- Ttmp[i,j,k] / sum(Ttmp[i,1:nCups,k])

Etmp[i,j,k] <- exp(-lambda[k] * distanceEgo[i,j])

Ep[i,j,k] <- Etmp[i,j,k] / sum(Etmp[i,1:nCups,k])

}

Ctmp[i,j] <- exp(-lambda[4] * distanceCenter[i,j])

Cp[i,j] <- Ctmp[i,j] / sum(Ctmp[i,1:nCups])

P[1,i,j] <- 1/12 #Guessing

P[2,i,j] <- Preference[j] #Cup Preference

P[3,i,j] <- Tp[i,j,3]*w[3] + Ep[i,j,3]*(1-w[3]) #Mixing

P[4,i,j] <- Cp[i,j] #Combination

}

*#This final step connects the probability calculations above to the data with a categorical distribution.*

responseCup[i] ~ dcat(P[theModel,i,1:nCups])

}

}
