# Supplementary material for: Four-Year-Olds Use a Mixture of Spatial Reference Frames
Source: PLoS One. 2015 Jul 2;10(7):e0131984. doi: 10.1371/journal.pone.0131984 (PMC4489865; doi:10.1371/journal.pone.0131984)
Supplement: S5 File — (DOCX) [file pone.0131984.s006.docx]

In the main text, we report the parameter estimates for *w* and λ when the Cue Mixing model is applied to children at each age (3, 4, 5, and 6 years old) and the prior on *w* is widened to (0,1). The estimates for both parameters rise, suggesting that children use the intrinsic frame more often and also become more accurate with whichever frame they choose. Here we look at the results from using the Cue Combination model instead.

**Figure S5-A. Parameter estimates from the Cue Combination model at each age.** This model has a separate parameter for the weight of each frame of reference (x axis) and the concentration of responses around the place indicated by their weighted average (y axis). As age increases, the intrinsic frame is given more weight (rightwards) and precision increases (upwards).

Results are broadly similar – *w* is very low at 3 years and very high at 6 years, and λ tends to increase with age. The main difference is at 5 years old, where *w* is estimated to be very high (95% CI from .85 to .99, versus .48 to .77 for Cue Mixing) and λ is estimated to be somewhat lower. This may just be due to the fact that the Cue Combination model is poorly fit in general to the 5 year olds (S1 File).
